# The impact of anastomotic leak on long-term oncological outcomes after low anterior resection for mid-low rectal cancer: extended follow-up of a randomised controlled trial

**DOI:** 10.1007/s00384-022-04204-9

**Published:** 2022-06-30

**Authors:** Quoc Riccardo Bao, Gianluca Pellino, Gaya Spolverato, Angelo Restivo, Simona Deidda, Giulia Capelli, Cesare Ruffolo, Francesco Bianco, Dajana Cuicchi, Elio Jovine, Raffaele Lombardi, Claudio Belluco, Antonio Amato, Filippo La Torre, Corrado Asteria, Aldo Infantino, Tania Contardo, Paola Del Bianco, Paolo Delrio, Salvatore Pucciarelli

**Affiliations:** 1 General Surgery 3, Department of Surgical, Oncological and Gastroenterological Sciences, University of Padova, Via Giustiniani 2, 35128 Padova, Italy; 2 Department of Advanced Medical and Surgical Sciences, Università Degli Studi Della Campania “Luigi Vanvitelli”, Naples, Italy; 3 Colorectal Surgery Unit, A.O.U. Cagliari, Department of Surgical Science, University of Cagliari, Cagliari, Italy; 4 Department of Abdominal Oncology, Istituto Nazionale Tumori – IRCCS Fondazione G. Pascale, Naples, Italy; 5 General Surgery Unit, Department of Alimentary Tract, IRCCS Azienda Ospedaliera Universitaria Di Bologna, Bologna, Italy; 6 General Surgery and Emergency, IRCCS Azienda Ospedaliera Universitaria Di Bologna, Bologna, Italy; 7 Department of Surgical Oncology, National Cancer Institute, Aviano, PN Italy; 8 Department of Coloproctology, Sanremo Hospital, Sanremo, IM Italy; 9 Division of Emergency and Trauma Surgery, Emergency Department, Policlinico Umberto I, College of Medicine and Dentistry, Sapienza University, Rome, Italy; 10 Department of General Surgery, Ospedale Carlo Poma, Mantua, Italy; 11 Surgical Unit, Department of General Surgery, Santa Maria Dei Battuti Hospital, San Vito al Tagliamento (PN), Italy; 12 Department of Surgery, Regional Centre for Laparoscopic and Robotic Surgery, Camposampiero Hospital, Padua, Italy; 13 Clinical Research Unit, Istituto Oncologico Veneto IOV – IRCCS, Padua, Italy; 14 Department of Colorectal Surgical Oncology, Istituto Nazionale Tumori – IRCCS Fondazione G. Pascale, Naples, Italy

**Keywords:** Rectal cancer, Anastomotic leak, Low anterior resection, Survival, Total mesorectal excision

## Abstract

**Purpose:**

The impact of anastomotic leaks (AL) on oncological outcomes after low anterior resection for mid-low rectal cancer is still debated. The aim of this study was to evaluate overall survival (OS), disease-free survival (DFS), and local and distant recurrence in patients with AL following low anterior resection.

**Methods:**

This is an extension of a multicentre RCT (NCT01110798). Kaplan–Meier method and the log-rank test were used to estimate and compare the 3-, 5-, and 10-year OS and DFS, and local and distant recurrence in patients with and without AL. Predictors of OS and DFS were evaluated using the Cox regression analysis as secondary aim.

**Results:**

Follow-up was available for 311 patients. Of them, 252 (81.0%) underwent neoadjuvant chemoradiotherapy and 138 (44.3%) adjuvant therapy. AL occurred in 63 (20.3%) patients. At a mean follow-up of 69.5** ± **31.9 months, 23 (7.4%) patients experienced local recurrence and 49 (15.8%) distant recurrence. The 3-, 5-, and 10-year OS and DFS were 89.2%, 85.3%, and 70.2%; and 80.7%, 75.1%, and 63.5% in patients with AL, and 88.9%, 79.8% and 72.3%; and 83.7, 74.2 and 62.8%, respectively in patients without (*p* = 0.89 and *p* = 0.84, respectively). At multivariable analysis, AL was not an independent predictor of OS (HR 0.65, 95%CI 0.34–1.28) and DFS (HR 0.70, 95%CI 0.39–1.25), whereas positive circumferential resection margins and pathological stage impaired both.

**Conclusions:**

In the context of modern multimodal rectal cancer treatment, AL does not affect long-term OS, DFS, and local and distant recurrence in patients with mid-low rectal cancer.

## Introduction

Rectal cancer represents a major cause of morbidity and mortality worldwide, accounting for about 736,000 new estimated cases and 340,000 estimated deaths in 2020 [1]. The standard of care for locally advanced mid-low rectal cancer is surgical resection with total mesorectal excision (TME) associated with neoadjuvant chemoradiotherapy (nCRT) [2].

Anastomosis-related complications represent the main source of morbidity after TME. Anastomotic leak (AL) occurs in up to 20% of low anterior resection (LAR) [3], and may require interventional treatment and, eventually, the need for a temporary or permanent stoma [4]. Several studies investigated risk factors for AL, and no differences were found using different reconstructive techniques [5]. In pre-multimodal treatment era, sex, obesity, and distance of the anastomosis from the anal verge were recognized as independent risk factors for AL after rectal resection [6]. Recently, many retrospective studies reported an increased rate of AL following nCRT [7–9]; nevertheless, results from randomised controlled trials (RCT) showed no difference in AL rate between nCRT and non-nCRT patients [5, 10, 11]. Regardless from these uncertainties, it is widely accepted that AL results in an increased overall postoperative morbidity and mortality, and prolonged in-hospital length-of-stay.

The impact of AL on oncological long-term outcomes have been previously investigated, with various results. The presence of a defect of the intestinal wall at anastomosis may promote the spilling of neoplastic cells and increase the risk of local recurrences. Furthermore, local inflammation caused by AL was found to promote the upregulation of receptors associated with adhesion of tumour cells [12, 13]; finally, the occurrence of an AL delays the beginning of adjuvant therapy, preventing an optimal local and distant control of the disease, resulting in a decreased disease-free survival (DFS) and overall survival (OS). In the meta-analyses by Mirnezami et al. and by Ha et al., AL following colorectal resection was reported to affect local recurrence and OS [14, 15]. More recently, other meta-analyses investigated specifically survival in patients with AL after rectal resection, reporting that AL had an adverse impact on survival and local recurrence [16–18]. Adverse effects of AL were also reported in a long-term analysis of series including patients with previous nCRT [19]. Other retrospective and observational studies, however, reported no impact of AL on long-term oncological outcomes [20–23].

Considering a potential adverse effect on recurrence, OS and DFS, the aim of the current study was to evaluate the impact of AL following LAR on OS, DFS, and local and distant recurrence, in patients enrolled in a multicentre RCT [5].

## Methods

### Study design

The present study represents an extended follow-up secondary analysis of a prospective multicentre RCT (NCT01110798) that enrolled patients affected by mid-low rectal cancer undergoing curative-intent surgery at 16 Italian centres between October 2009 and February 2016 [5, 24]. Long-term survival analyses were originally designed as secondary outcome of the trial. Inclusion criteria of the trial were the following: age > 18 years, biopsy-proven adenocarcinoma of the rectum up to 11 cm from the anal verge, resectable disease with LAR and stapled anastomosis, and curative-intent resection (R0-R1). Exclusion criteria were non-curative resection (R2), metastatic disease, previous history of colonic resection, and handsewn coloanal anastomosis.

The study was first approved by the Ethics Committee of the coordinating centre and subsequently approved by the local committee of every participating centre. Patients provided written informed consent to participate in this study. The current analysis was reported following the The Strengthening the Reporting of Observational Studies in Epidemiology (STROBE) Statement [25].

### Endpoints and outcome measures

The primary endpoint on this study was the impact of AL on long-term oncologic outcomes. The outcome measures were OS, DFS, and incidence of local and distant recurrence. The secondary endpoints included the rate and pattern of recurrence, and factors associated with oncologic outcomes.

### Data of interest and treatment details

The following data were recorded for each patient: gender, age, American Society of Anaesthesiologists (ASA) score, Eastern Cooperative Oncology Group (ECOG) performance status scale, Body Mass Index (BMI), distance of the tumour from the anal verge, baseline Carcinoembryonic Antigen (CEA) level, clinical staging, and nCRT regimen. The race/ethnicity distribution of the enrolled patients was not available, since it was not collected in the original study. All the patients underwent a LAR with standard TME [26], and were randomised to receive either a stapled colonic J-Pouch or a straight colorectal anastomosis. The creation of a covering stoma was mandatory. Long-course preoperative CRT (capecitabine-based chemotherapy + 45-–50.4-Gy radiotherapy) or short-course radiotherapy (5 Gy in 5 fractions) was administered as recommended in national guidelines [27]. Adjuvant treatment was offered to patients with pTNM II stage with high-risk features, or pTNM III stage. In patients who underwent adjuvant treatment, stoma reversal was recommended after the completion of the treatment.

### Definitions

Major non-anastomotic complications were defined as any complication requiring interventional treatment (i.e. surgical or radiological procedures, Clavien-Dindo grade ≥ 3a [28]). According to the International Study Group of Rectal Cancer, AL was defined as any defect of the anastomosis leading to a communication between intra- and extra-luminal compartments. Leak originating from suture or staple line of the colonic J-Pouch and pelvic abscess in the proximity of the anastomosis were considered as AL [29]. According to the study protocol, the evaluation of anastomotic integrity was mandatory within 30 days of the index surgery and was assessed by endoscopic or radiologic (i.e. soluble contrast medium enema/CT) investigation. The severity of AL was classified according to the definition and grading system proposed by Rahbari et al. [29]*.* Grade A was defined as AL resulting in no change in patient’s management, grade B as AL requiring therapeutic intervention without re-laparotomy, and grade C as AL requiring re-laparotomy.

For every patient, pathological data were collected, including tumour size and grading, circumferential resection margin (CRM) status, pathologic tumour (pT) and nodal (pN) stage, number of harvested lymph nodes, and radicality of resection (R0-R1). Clinical and pathological TNM staging were reported according to the American Joint Committee on Cancer 8th Edition [30].

### Long-term outcomes definition

Patients underwent a standard oncological follow-up, according to national guidelines [27]. Physical examination, routine blood tests, and CEA were performed every 4 months in the first 2 years, and then every 6 months for a total of 5 years. Chest-abdominal CT was performed every 6 months for 5 years. Colonoscopy was recommended 1 year after surgery. For the purpose of this study, every participating centre was contacted to update the oncological outcomes (date of death or last follow-up, and date of local or distant recurrence). Local recurrence was defined as any pelvic endoluminal or extra-luminal recurrence, while recurrences outside the pelvis were defined as distant.

### Statistical analysis

Continuous data are expressed as mean value with standard deviation. Significant differences between the two groups were test by the Fisher exact test for categorical variables, and independent sample *t* test for continuous variables. To estimate the OS and DFS, the Kaplan–Meier method and log-rank test were used to compare the survival of the groups. Each outcome was calculated from the date of surgery to the date of the event (local or distant recurrence, death, or the last follow-up). Multivariable survival analysis for OS and DFS was performed using Cox regression model. Results are reported as Hazard Ratio with 95% confidence intervals (CI). All analyses were carried out with STATA version 13.0 (StataCorp, College Station, TX).

## Results

### Patients, tumour, and treatment characteristics

Eleven out of 16 centres agreed to participate to this study, resulting in the inclusion of long-term outcomes of 311 out of 379 patients who were initially enrolled in the trial.

The following baseline clinical stage was available in 308 patients: cT1 (*n* = 8, 2.6%), cT2 (*n* = 43, 14%), cT3 (*n* = 249, 81.0%), and cT4 (*n* = 8, 2.6%). Lymph nodes were found to be clinically positive in 192 (63.0%) patients. A total of 252 (81.0%) patients underwent preoperative treatment, 46 (14.8%) underwent a short-course radiotherapy, 204 (65.6%) a long-course nCRT, and two underwent chemotherapy only. In patients who received nCRT, the most common chemotherapy regimens included capecitabine/5-FU alone (*n* = 139, 44.7%), or in associations with oxaliplatin (*n* = 13, 4.2%).

A conventional open LAR was performed in 200 (64.3%) patients, while a minimally invasive approach was performed in 111 (35.7%). Overall, 145 (46.6%) patients underwent a colonic J-pouch, and 166 (53.3%) a straight colorectal anastomosis. The mean distance of the anastomosis from the anal verge was 4.3 ± 1.5 cm. The mean postoperative hospital length-of-stay was 10.7 ± 10.2 days. Postoperative not AL-related complications were found in 94 (30.2%) patients; of these, 54 (17.4%) were minor and 40 (12.9%) were major. Readmission was required in 25 (8.0%) patients, and re-operation in 21 (6.8%).

### Anastomotic leak

Patients, tumour, and treatment characteristics of the study group according to the presence or absence of AL are summarised in Table 1. Sixty-three (20.3%) patients were found to have an AL. Twenty (6.4%) patients had grade A, 31 (10.0%) grade B, and 12 (3.9%) grade C AL. Most of the patients with AL (*n* = 54, 85.7%) had received nCRT. AL was more common in men than in women (24.3% vs 14.3%, *p* = 0.03). ASA score and BMI were higher in the AL than in non-AL group (*p* = 0.03 and *p* = 0.01 respectively). The mean distance of the tumour from the anal verge was 7.3 ± 2.2 cm and was shorter in patients with AL than in those without AL (6.7 ± 2.3 vs 7.5 ± 2.1 cm, *p* = 0.008). Pathological stage, rate of CRM infiltration, number of lymph nodes harvested, and rate of patients who underwent nCRT and adjuvant chemotherapy did not differ between patients with and without AL (Table 1). The postoperative hospital length-of-stay was longer in patients with AL than in those without AL (median (IQR) 9(7–14) vs 8(7–11) days, *p* = 0.001).

**Table 1:** Patients’ characteristics

			**Anastomotic leak**		
**Variables**		**Overall**	**Yes**	**No**	***p*-value**
**Age**	Mean ± SD	63.6 ± 10.9	64.7 ± 11.1	63.4 ± 10.8	0.39
**Gender, *n* (%)**	Men	185 (59.5)	45 (71.4)	140 (56.4)	**0.03**
	Women	126 (40.5)	18 (28.6)	108 (43.5)	
**ASA score, *n* (%)**	1	113 (36.3)	19 (30.2)	94 (37.9)	**0.03**
	2	143 (45.9)	25 (39.7)	118 (47.6)	
	3	53 (17.0)	18 (28.6)	35 (14.1)	
	4	2 (0.6)	1 (1.6)	1 (0.4)	
**ECOG PS, *n* (%)**	0	176 (60.1)	34 (58.6)	142 (60.4)	0.55
	1	92 (31.4)	17 (29.3)	75 (31.9)	
	2	20 (6.8)	5 (8.6)	15 (6.4)	
	3	4 (1.4)	2 (3.5)	2 (0.9)	
	4	1 (0.3)	0	1 (0.4)	
**BMI**	Mean ± SD	25.77 ± 4.3	26.95 ± 5.5	25.47 ± 3.9	**0.01**
**Smoker**	Yes	73 (23.5)	15 (23.8)	58 (23.4)	0.9
	No	238 (76.5)	48 (76.2)	190 (76.6)	
**LOS**	Median (IQR)	8 (7–11)	9 (7–14)	8 (7–11)	**0.001**
**Distance from a.v**	Mean ± SD	7.3 ± 2.2	6.7 ± 2.3	7.5 ± 2.1	**0.008**
**CEA**	Mean ± SD	7.0 ± 25.4	8.3 ± 33.4	6.7 ± 23.1	0.65
**Pathological stage, *n* (%)**	0	43 (13.8)	9 (14.3)	34 (13.7)	0.96
	1	122 (39.3)	23 (36.5)	99 (39.9)	
	2	64 (20.6)	14 (22.2)	50 (20.2)	
	3	82 (26.4)	17 (27.0)	65 (26.2)	
**CRM, n (%)**	-	298 (97.4)	58 (95.1)	240 (98.0)	0.199
	+	8 (2.6)	3 (4.9)	5 (2.0)	
**Harvested lymph nodes**	Mean ± SD	17.8 ± 9.7	18.7 ± 12.0	17.5 ± 9.1	0.422
**nCRT, *n* (%)**	No	59 (19.0)	9 (14.3)	50 (20.2)	0.288
	Yes	252 (81.0)	54 (85.7)	198 (79.8)	
**Adjuvant therapy, *n* (%)**	No	173 (55.6)	40 (63.5)	133 (53.6)	0.159
	Yes	138 (44.4)	23 (36.5)	115 (46.4)	

*ECOG PS* Eastern Cooperative Oncology Group Performance Status, *BMI* body mass index, *LOS* length-of-stay, *A.V.* anal verge, *CEA* carcinoembryonic antigen, *CRM* circumferential resection margin, *nCRT* neoadjuvant chemoradiotherapy

### Long-term outcomes and pattern of recurrence

At a mean follow-up of 69.5 (31.9) months, recurrence occurred in 61 (19.6%) patients. Of these, 12 (3.9%) patients developed local recurrence, 38 (12.2%) distant recurrence, and 11 (3.5%) both local and distant recurrence. The most common sites of recurrence were lungs (*n* = 33, 10.6%) and liver (*n* = 22, 7.1%). The mean time from surgery to local and distant recurrence was 26.0 ± 19.3 and 29.5 ± 23.4 months, respectively.

The pathological stage of 63 patients with AL was the following: pT0 (*n* = 9, 14.3%), pT1 (*n* = 10, 15.8%), pT2 (*n* = 20, 31.7%), pT3, (*n* = 23, 36.5%), and pT4 (*n* = 1, 1.5%). In the same patients, 46 (73.0%) were found to be pN0. The final pTNM stage was the following: stage 0 (*n* = 9, 14.3%), stage I (*n* = 23, 36.5%), stage II (*n* = 14, 22.2%), and stage III (*n* = 17, 27.0%). Adjuvant chemotherapy was performed in 23 (36.5%) patients, 16 of whom have had a clinically relevant AL (grades II–III).

Among the patients who experienced recurrence, 10 (16.3%) also experienced an AL (grade A *n* = 2, grade B *n* = 4, grade C *n* = 4). In these patients, the recurrences were local (*n* = 1), distant (*n* = 4), and both local and distant (*n* = 5). The mean time to local and distant recurrence was 25.7 ± 14 and 28.2 ± 19.3 months, respectively.

Among the 20 patients with a grade A AL, 2 (10.0%) patients experience recurrence (distant only, *n* = 1, local + distant, *n* = 1), and 3 of them deceased, only 1 for progression disease. Out of 31 patients with a grade B AL, 4 (12.9%) patients experience recurrence (local only, *n* = 1, distant only, *n* = 2, and local + distant *n* = 1), and 8 of them deceased, only 2 for progression disease. Among 12 patients with a grade C AL, 4 (33.3%) patients experienced recurrence (distant only, *n* = 1, and local + distant *n* = 3), and 3 of them deceased, 2 for progression disease.

### Primary aim: AL and long-term oncologic outcomes

**Fig. 1:**
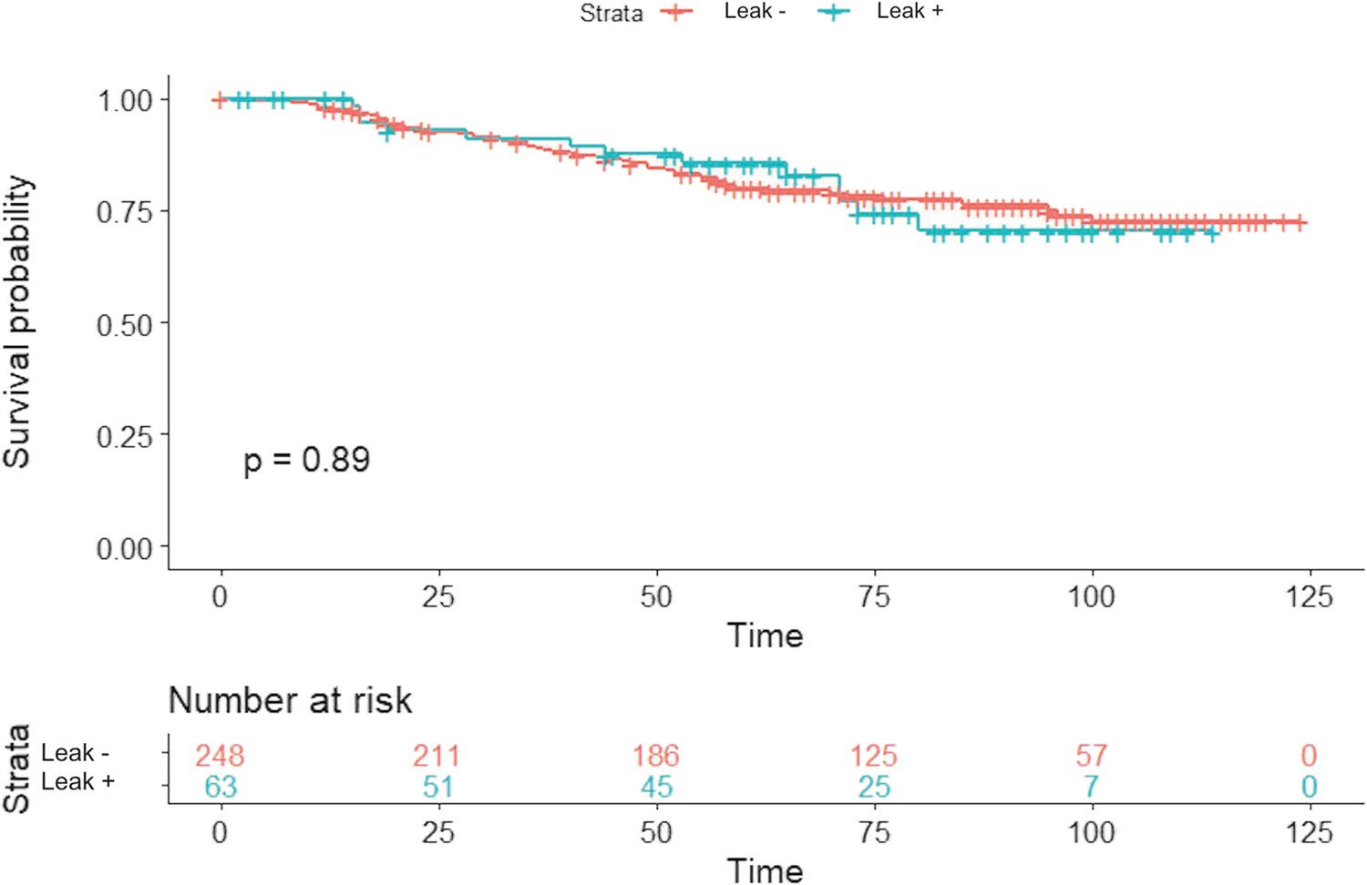
Kaplan–Meier overall survival estimate according to anastomotic leak. 3-, 5-, and 10-year overall survival was 89.2%, 85.3%, and 70.2% vs 88.9%, 79.8%, and 72.3% in patients with anastomotic leak and without anastomotic leak respectively (log-rank test *p* = 0.89)

**Fig. 2:**
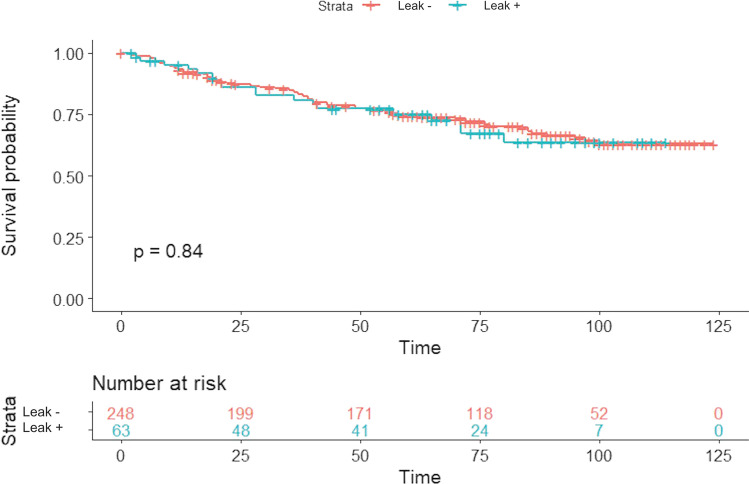
Kaplan–Meier Disease-Free Survival estimate according to anastomotic leak. 3-, 5-, and 10-year disease-free survival was 80.7%, 75.1%, and 63.5% vs 83.7%, 74.2%, and 62.8% in patients with anastomotic leak and without anastomotic leak respectively (log-rank test *p* = 0.84)

In patients with AL, the estimated cumulative 3-, 5- and 10-year OS were 89.2%, 85.3%, and 70.2%, respectively, whereas the estimated cumulative 3-, 5- and 10-year DFS were 80.7%, 75.1%, and 63.5%, respectively. Comparing these curves with those of patients without AL, no differences were found between the two groups (*p* = 0.89, and *p* = 0.84 respectively) (Figs. 1 and 2).

**Fig. 3:**
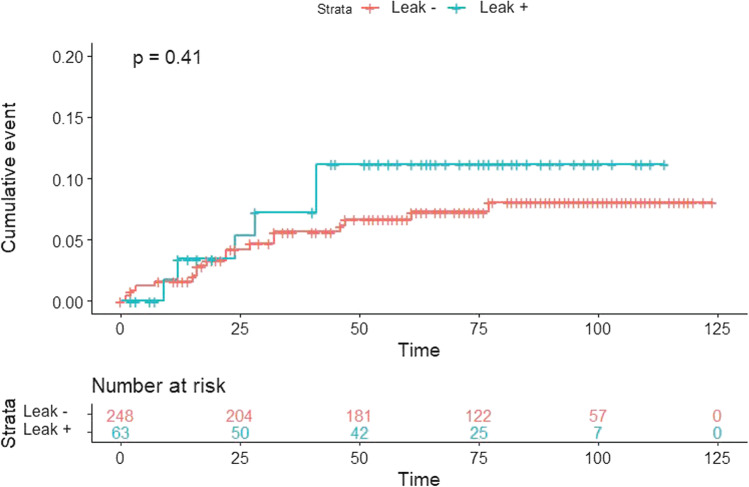
Local recurrence according to anastomotic leak

**Fig. 4:**
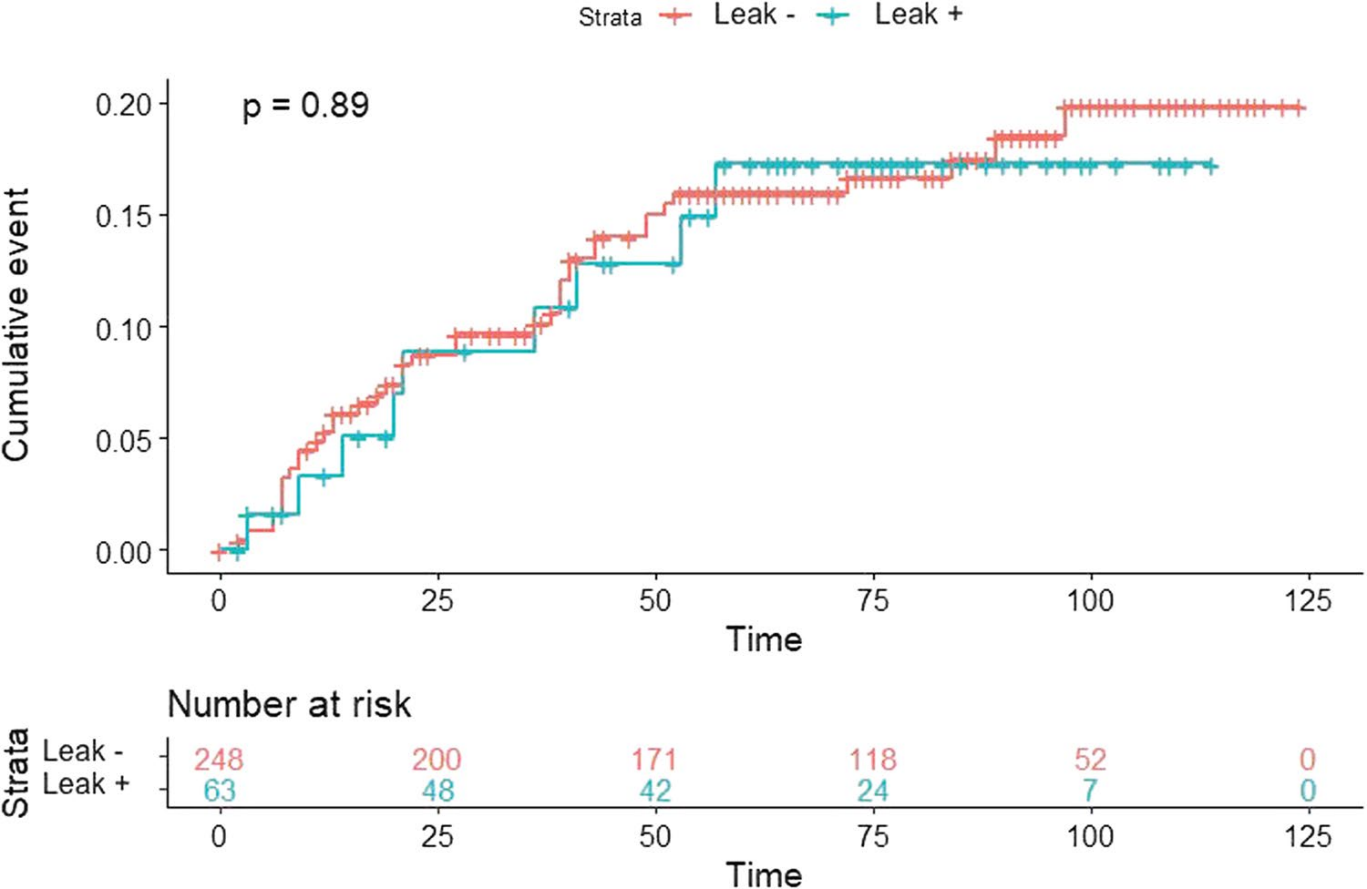
Distant recurrence according to anastomotic leak

No differences between patients with and without AL were found in terms of local and distant recurrence (*p* = 0.41, and *p* = 0.89 respectively) (Figs. 3 and 4).

### Secondary aim: predictors of survival

On multivariable analysis (Table 2), preoperative CEA level (HR 1.01. 95%CI 1.00–1.01, *p* = 0.02), distance from the anal verge (HR 0.89, 95%CI 0.79–1.00, *p* = 0.05), pT stage (HR 1.43, 95%CI 1.03–1.99, *p* = 0.033), pN stage (HR 1.64, 95%CI 1.11–2.42, *p* = 0.014), and a positive CRM (HR 4.44, 95%CI 1.52–12.9, *p* = 0.006) were found to be independent predictors of OS.

**Table 2:** Multivariable analysis to identify independent predictors of survival

**Variables**		**OS** **HR (95%CI)**	***p*-value**	**DFS** **HR (95%CI)**	***p*-value**
Gender	Male	1.34 (0.76–2.36)	0.3	1.13 (0.70–1.83)	0.6
Age		1.01 (0.98–1.04)	0.5	1.00 (0.98–1.03)	0.7
nCRT	Yes	1.73 (0.64–4.69)	0.3	1.87 (0.81–4.33)	0.15
Adjuvant therapy	Yes	0.70 (0.37–1.32)	0.3	0.62 (0.35–1.10)	0.10
CEA		1.01 (1.00–1.01)	**0.02**	1.02 (1.01–1.02)	** < 0.001**
ASA score		1.31 (0.87–1.97)	0.2	1.13 (0.80–1.61)	0.5
Distance from the a.v		0.89 (0.79–1.00)	**0.05**	0.92 (0.83–1.02)	0.12
cT		1.13 (0.53–2.43)	0.8	1.12 (0.57–2.20)	0.7
cN		1.11 (0.63 – 1.96)	0.7	1.11 (0.69, 1.81)	0.7
pT		1.43 (1.03 – 1.99)	**0.033**	1.33 (1.01, 1.74)	**0.045**
pN		1.64 (1.11 – 2.42)	**0.014**	1.76 (1.24, 2.49)	**0.002**
CRM	+	4.44 (1.52–12.9)	**0.006**	4.96 (1.90–12.9)	**0.001**
Anastomotic leak	Yes	0.65 (0.34–1.28)	0.2	0.70 (0.39–1.25)	0.2
Grading		0.96 (0.67–1.39)	0.8	0.94 (0.68–1.29)	0.7

Preoperative CEA level (HR 1.02 95%CI 1.01–1.02, *p* < 0.001), pT stage (HR 1.33, 95%CI 1.01–1.74, *p* = 0.045), pN stage (HR 1.76, 95%CI 1.24–2.49, *p* = 0.002), and a positive CRM (HR 4.96, 95%CI 1.90–12.9, *p* = 0.001) were found to be independent predictors of DFS.

AL was not an independent predictor of OS (HR 0.65, 95%CI 0.34–1.28, *p* = 0.2) nor of DFS (HR 0.70, 95%CI 0.39–1.25, *p* = 0.2).

## Discussion

This study aimed to investigate whether AL following LAR for mid-low rectal cancer may have a negative impact on oncological outcomes, focusing on OS and DFS. AL did not impact OS, DFS, and local and distant recurrence, but other factors including tumour-related characteristics and surgical clearance did.

Over the last decades, several studies have looked at a potential correlation between AL and long-term outcomes after rectal cancer surgery, but results are still controversial, because of several confounders and factors that need to be considered in these patients. The findings of the current study are in line with those of Crippa et al. from the Mayo Clinic, who found a local recurrence rate of 4.8% in patients with AL, which was not different compared to patients without AL. Moreover, AL was not found to be an independent predictor of local recurrence [21]. Similar findings were previously found by Smith et al. who reported the Memorial Sloan Kettering Cancer Center experience. Even if a clinical AL was associated with a delay of adjuvant treatment administration, it was not associated with an increased local recurrence rate. Again, the AL was not found to be an independent risk factor for local recurrence [23]. The same considerations apply for OS [21, 23]. In the present study, the survival outcomes in terms of OS and DFS did not differ between patients with and without AL. In a more recent RCT (COLOR II) on 764 patients with rectal cancer randomised to laparoscopic versus open surgery, AL was independently associated with reduced DFS and higher risk of local recurrence, but not with OS and distant recurrence [31].

A significant number of meta-analyses have been published on this topic, using data from retrospective and prospective studies [14–18], and the more recent did found a correlation between AL and survival and local recurrence [16, 17]. However, the significant heterogeneity between the included studies, the wide time frames of treatment, and the inclusion of studies on patients who did not receive neoadjuvant treatments make it difficult to draw definitive conclusions.

Apart from tumour stage and CEA levels, an important factor to consider when it comes to assessing long-term outcomes and disease control after curative LAR for cancer, is whether the patients underwent concomitant treatments. Jang et al. reported on 698 patients with locally advanced mid-low rectal cancer treated with long-course nCRT followed by TME [22], with an AL rate of 6.7%. Most of these AL were clinically severe and required re-operation (grade C = 83%), resulting in a statistically significant delay in adjuvant therapy in patients with AL compared with those without AL. However, survival analysis reported no differences concerning the 5-year OS (90.9% vs 86.2%, *p* = 0.242) and DFS (93.3% vs 94.9%, *p* = 0.653). An analysis of the prospective Spanish Rectal Cancer Project Registry on 1153 patients reported a clinically relevant AL rate of 9.4% after LAR, which was not an independent predictor of OS (HR 1.10 95%CI 0.73–1.65; *p* = 0.648) nor cancer-specific survival (HR 1.23 95%CI 0.75–2.02; *p* = 0.421) [20]. However, a retrospective propensity-score analysis by Kulu et al. found AL to be an independent risk factor for worse OS and DFS [32]. Of note, only 50% of their patients received nCRT and 25% received adjuvant therapy. The long-term survival analysis of the German Rectal cancer trial found that AL was associated with impaired 10-year OS (51.0% vs 65.2%, *p* = 0.02) in all treatment group. However, the rate of distant metastases and local recurrence was higher after AL only in those patients who did not receive neoadjuvant or adjuvant CRT, even in stage I cancers [19]. The association with chemotherapy and radiotherapy, either before or after surgery, seems therefore to play a relevant role on the outcomes. Most patients in the current study received concomitant therapy. Overall, 81% and 44% of the patients underwent nCRT and adjuvant therapy respectively, and more than 1/3 of the patients (37.0%) to both neoadjuvant and adjuvant therapy, further advocating a protective effect in term of long-term outcomes irrespective from if AL occurs, but neither nCRT nor adjuvant therapy were protective factors for OS and DFS at regression analysis.

Finally, another relevant aspect that needs attention is the importance of performing adequate, radical surgery during LAR, achieving complete oncological clearance. Patients with R2 resection were not included in the study, and a microscopically positive CRM was the strongest predictor of worse survival, increasing the risk of shorter OS and DFS by more than 4 times (Table 2). The surgeon factor should therefore not be underestimated [33], as a proper oncological resection remains the most important prognosticator of survival.

## Study limitations and strengths

This study does have limitations. Some data concerning the adjuvant therapy (e.g. regimen, date of start, potential delay) were not consistently captured. Also, not all centres joined the extended follow-up analyses, reducing the available sample of the patients included. As matter-of-fact, data in only 11 out of 16 centres, resulting in 311 out of 379 patients initially enrolled in the trial, were available. The study sample size of was estimated on the primary outcome of the RCT, and the trial was interrupted at the second ad interim analysis. Of course, by interrupting the RCT and reducing the sample size, the risk of type 2 statistical error on this secondary outcome is increased. However, by considering the size of the population with an AL, setting the power of the study at 0.80, the estimable difference in survival is up to 20% approximately.

However, published studies have several limitations that were removed in the current analysis. The most common shortcomings of the previous reports include the retrospective design, the lack of a shared and validated definition and grading of AL, the inclusion of both colon and rectal cancer resections, a wide inclusion of patients not treated with neoadjuvant or adjuvant treatment, and the lack of data regarding neoadjuvant and adjuvant treatment. On the contrary, the current study included long-term analysis as scheduled secondary outcome of a multicentre RCT. The anastomosis was systematically evaluated after surgery, and events were reported according to a shared and validated definition and grading system [29], and the overall AL rate is high. However, due to the low rate of recurrences in grades A, B, and C, AL is difficult to assess a statistical difference, even if the rate is relatively higher in most severe leakage (grades A, B, and C 10.0, vs 12.9, vs 33.3% respectively). Additional strengths of this study include its multicentric design, the long-term follow-up, the prospective collection of data, and the inclusion of mid and low rectal cancer only. Furthermore, most of the patients (80%) underwent nCRT, and 45% adjuvant treatment in high-risk stage II and stage III disease, making results easily generalizable.

Of note, patients in the current study who experienced an AL were promptly treated at tertiary centres, thereby optimising the chances of long-term success—potentially reducing the long-term impact on survival.

## Conclusions

In patients with mid-low rectal cancer who undergo LAR in the context of a multidisciplinary team and multimodal management, AL do not impair OS, DFS, and do not increase local and distant recurrence rates.

Pathological stage, preoperative CEA levels, and CRM status are independent predictors of long-term survival. This suggests the importance of adequate preoperative planning, and the relevance of following the oncological principles of radical surgery. On the other hand, the study allows to reassure patients who experience AL that they should not expect worse long-term outcomes following such a debilitating complication.

## Data Availability

The dataset used in the current study is available on reasonable request to the authors.
